# Effectiveness of acupuncture at acupoint BL1 (Jingming) in comparison with artificial tears for moderate to severe dry eye disease: a randomized controlled trial

**DOI:** 10.1186/s13063-022-06486-4

**Published:** 2022-07-27

**Authors:** Xue Zhang, Bo Zhang, Siyang Peng, Guoliang Zhang, Jumei Ma, Wenzeng Zhu

**Affiliations:** 1 Department of Acupuncture, South Area of Guang’anmen Hospital, China Academy of Chinese Medical Sciences, No. 138 Xingfeng Street, Daxing District, Beijing, 102618 China; 2 Department of Acupuncture, Guang’anmen Hospital, China Academy of Chinese Medical Sciences, Beijing, 100053 China; 3 Department of Ophthalmology, Guang’anmen Hospital, China Academy of Chinese Medical Sciences, Beijing, 100053 China; 4 Department of Ophthalmology, South Area of Guang’anmen Hospital, China Academy of Chinese Medical Sciences, Beijing, 102618 China

**Keywords:** Dry eye, Acupuncture, RCT, Artificial tears, SIT

## Abstract

**Background:**

The global incidence of dry eye disease (DED) is very high. DED seriously affects the quality of life of patients; however, the current curative effect of medicine for moderate to severe DED is poor. This randomized clinical trial was planned to investigate the effect of acupuncture compared with artificial tears (AT) on moderate to severe DED.

**Methods:**

A randomized clinical trial was performed at 2 hospitals in China. 120 DED patients were randomly equally divided into an acupuncture and an artificial tear group. Either acupuncture or artificial tears was performed for an 8-week period, and a 24-week follow-up was performed. The primary outcome measure was the Schirmer-I test (SIT) change from baseline. The secondary outcome measures included the numerical rating scale (NRS) change from baseline for improvement in ocular symptoms, the ocular surface disease index (OSDI), the tear-film break-up time (TBUT), corneal fluorescein staining (CFS), and acupuncture acceptability. Adverse events also were monitored and documented.

**Results:**

For the primary outcome, the mean changes from baseline in the SIT values were significantly different between the acupuncture (5.75 [2.53–9.75]) and AT (0.52 [− 1.18–2.46]) groups at week 8 with a between difference of 5.23 (*P* < 0.05). Between-group differences of 8.49 in OSDI score change from baseline differed significantly at week 8 (*P* < 0.05). However, between-group differences of the changes in the average symptom NRS score, TBUT, and CFS did not differ significantly at week 8. Five cases experienced acupuncture-related adverse events.

**Conclusions:**

This randomized clinical trial found that acupuncture at BL1 significantly promoted tear secretion. Acupuncture showed greater benefits than AT for moderate to severe DED. However, the study findings warrant verification.

**Trial registration:**

Registration number: ChiCTR1800015831. Name of trial registry: Efficacy and safety of acupuncture in the treatment of moderate to severe dry eye disease: a randomized controlled trial. Registered on 23 April 2018 (https://clinicaltrials.gov/).

**Supplementary Information:**

The online version contains supplementary material available at 10.1186/s13063-022-06486-4.

## Background

Dry eye disease (DED) is a chronic ocular surface disease accompanied by symptoms of eye discomfort [1]. It refers to abnormal tear quality or quantity and dynamics, of any cause, resulting in tear-film instability and/or eye surface abnormalities. DED symptoms include eye dryness, foreign body sensation, pain, visual fatigue, and blurred vision. The global DED incidence varies (5–34%) [2], and is higher in Asian countries. Moreover, the incidence has increased with the recently increased exposure to display screens, abuse of eye medicines, and continuous improvement in DED diagnosis. DED causes include tear-film instability, tear hyperosmolality, ocular surface inflammation, injury, and neurosensory abnormalities [3]. It increases financial burden and reduces work efficiency, and quality of life [4–6].

Based on expert consensus [7], Artificial tears (ATs) are among the most commonly used drug treatments; ATs lubricate the eye surface, supplement tears, and reduce tear osmotic pressure, but only temporarily relieve DED symptoms. Effective treatment for severe DED is currently lacking. Non-drug treatments include surgery, lacrimal duct embolism, psychological counseling, physiotherapy, health guidance, wet chamber mirror, and silicone eye masks. Surgical treatment may have risks and side effects, making it a less acceptable treatment strategy. The efficacy of other therapies is not established; thus, these are not widely used [8].

Acupuncture therapy has a long history of use in China. Systematic reviews of randomized controlled trials (RCTs) have demonstrated that acupuncture is effective for DED [9–11]. However, previous studies [12, 13] showed that acupuncture treatment only improved subjective symptoms and lack the effectiveness of objective indicators for DED. Moreover, previous clinical studies of acupuncture generally included participants with mild to moderate disease, and the efficacy of acupuncture on moderate to severe DED remains uncertain [14]. Furthermore, the effect of only using periocular acupoints for treating DED was not significant based on a systematic review [15]. Thus, further studies with periocular acupoints accompanied by effective acupuncture manipulation are needed to investigate the effects for patients with moderate to severe DED. Although ATs are currently recommended as a conservative and effective treatment, they cannot replace naturally secreted tears as the lack of oxygen, vitamin A, mucoprotein, and other nutrients present in naturally secreted tears. In our previous clinical study [16], only acupuncture at the BL1 (Jingming) was effective in promoting tear secretion and improving DED symptoms.

In this RCT, we compared the effectiveness of acupuncture at BL1, with repeated needle manipulation until tear flow, with ATs for treating moderate to severe DED. We evaluated the improvements in objective and subjective indicators after an 8-week treatment period and a subsequent 24-week follow-up period.

## Methods

**Fig. 1:**
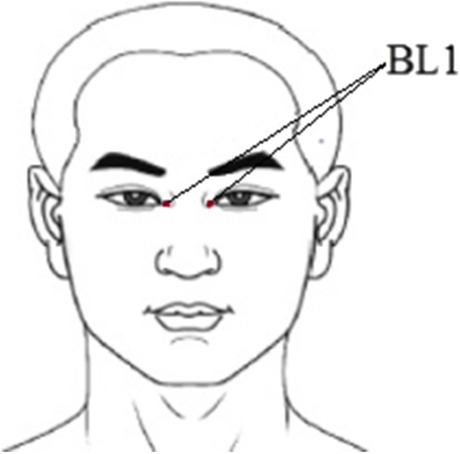
Location of acupuncture points

This 2-center, RCT was approved by the Ethics Committee of Guang’anmen Hospital of China Academy of Chinese Medical Sciences. The study commenced on September 1, 2018, and was completed on December 31, 2020. The flow chart is shown in Fig. 1. Before inclusion, each participant provided written informed consent. The study protocol has been published previously [17]. The original trial protocol and statistical analysis methods are available (Supplementary 1). The trial was conducted and reported in accordance with CONSORT guidelines.

### Study design

Patients were randomized to the acupuncture or AT groups based on a random distribution sequence. Clinical Laboratory of affiliated drugs of Guang’anmen Hospital was responsible for random grouping by computer. Patients were randomly assigned to the acupuncture or AT group using a block size of 4 stratified by centers, the final group assignments were placed in 120 opaque sealed envelopes, which were serially numbered and kept by a researcher not involved in treatment procedures or data analysis. After informed consent was obtained, the researcher opened the envelope according to the order in which the participant entered the trial and was provided for the prescribed treatment. All participants received either acupuncture or ATs 3 times per week over the 8-week period, and both groups were followed up for another 24 weeks.

### Study population

DED participants were recruited from advertisements posted by the hospital. Participants were eligible only if they were 18–60 years old and met the diagnostic criteria of moderate-to-severe DED [18, 19] : They were required to have one or more symptoms of DED (e.g. eye dryness, foreign body sensation, itching, burning, stinging, visual fatigue, photophobia or blurred vision ) with the severity of ≥4 (range: 0–10) measured by numerical rating scale (NRS). a Schirmer-I test (SIT) (with the application of cocaine, a local anesthetic) value < 10mm/5 min, a tear-film break-up time (TBUT) < 10 s, and corneal fluorescein staining (CFS) scale ≥ 1. Only the more severely affected eye was analyzed. Major exclusion criteria were a DED surgery history within 3 months, severe ocular diseases or immune system diseases, current use of contact lenses, and pregnancy or lactation. Participants were permitted to continue their other chronic treatments excluding DED.

### Intervention

All the participants must stop any treatment related to DED for a week before the trial commenced. In the trial, other drugs unrelated to this trial for DED couldn’t be used. Treatments were initiated after randomization immediately. Health education was provided for all participants before treatment. Participants were required to reduce their exposure to mobile phones. When they had to sustain reading or using their computers, it was recommended to rest for 5–10 min per hour. They were also required to sleep sufficiently every day. During the study, other treatments related to DED were not permitted to receive. when participants felt discomfortable, it was not recommended to buy drugs by themselves.

The treatment plan was derived from the consensus of acupuncturists and ophthalmologists based on a pilot trial. Acupuncture was administered at Jinming (BL1) in the acupuncture treatment group. BL1 is known as the tear hole acupoint and is associated with tear secretion. It is commonly used for treating eye diseases. Huatuo stainless-steel needles (0.3 × 40 mm, Suzhou Medical Appliance Factory, Suzhou, China) were used in the acupuncture group, in accordance with the STRICTA guidelines. Acupuncturists had at least 5 years of acupuncture experience and received training before administering treatments in this study. The training included detailing how to perform the acupuncture. The BL1 acupoint is located slightly above the inner canthus (Fig. 1) While the participant sat on a chair, the acupuncturist lifted the upper eyelid of the subject with the left hand, fully exposed the inner canthus of the eye, and punctured the acupoint with the needle, vertically, from the inner canthus of the eyes at approximately 0.5 inches. The needle was then removed and re-inserted vertically at this position, approximately every 3 s. After several insertions, tears flowed freely from the eye, at which point the acupuncturist removed the needle. Treatment was administered 3 times per week for 8 weeks.

The control group was treated with sodium hyaluronate eyedrops (EUSAN GmbH, Saarbrücken, Germany). Participants were asked to use them as needed (1 drop, 4 times per day, in the early morning, early afternoon, afternoon, and before bedtime), with an average interval of 6 hours, for 8 weeks consecutively. A diary recording the frequency of ATs used during the treatment period was collected at each visit.

### Outcome measurements

#### Primary outcome measure

The primary outcome was the increase from baseline in the value of the SIT, with anesthesia, at week 8. The SIT was performed using a tear secretion test paper (Color Bar™ Schirmer tear test, Eagle Vision, Inc., Memphis, TN, USA). Tear volume was measured by placing the strip down the outer third of the eyelid and measuring the length of the wet part of the test strip 5 min later (the SIT value). Although there is no clinical significance yet, the SIT value is considered an important indicator of DED severity and positively correlates with DED symptoms and quality of life [20].

#### Secondary outcome measures

Secondary outcomes included the following. The SIT value with anesthesia at baseline, week 4, and week 32 was measured, similarly to week 8.

The average DED symptom intensity of the past week, as assessed by the participant with a self-administered numerical rating scale (NRS) (range: 0–10), was based on well-known DED symptoms (eye dryness, foreign body sensation, itching, burning, stinging, visual fatigue, photophobia, blurred vision) at baseline, weeks 4, 8, and 32. The individual score and the mean of these 8 symptom scores (global symptom score) were calculated and compared.

The Ocular Surface Disease Index (OSDI) was assessed at baseline, weeks 4, 8, and 32. The OSDI is commonly used to evaluate the influence of ocular surface disease on patients’ daily life. The scale includes 3 aspects: eye symptoms, visual-related function, and environmental stimulus factors; the higher the OSDI score (range: 0–100), the more serious the impact of the disease on daily life. The change in the total scores reflected the overall improvement in the patients’ eye symptoms.

The tear break-up time (TBUT) was measured at baseline, weeks 4, 8, and 32. The TBUT test is used to assess tear-film stability and reflects different pathophysiologies according to rupture mode [21]. After staining with sodium fluorescein (2.5%), the time taken for the first dry spot on the tear-film was measured thrice with a slit lamp, and the average time was taken as the TBUT.

Corneal fluorescein staining (CFS), which was used to assess ocular surface damage as well as monitor the clinical response to therapy, was measured at baseline, weeks 4, 8, and 32. Corneal staining was examined under standard illumination with a slit lamp, using a cobalt blue filter, and was graded using the modified Oxford scale [22].

Acceptability of acupuncture or AT was evaluated at week 8, using a 4-point scale, with 1 representing “very difficult to accept,” 2 representing “difficult to accept,” 3 representing “easy to accept,” and 4 representing “very easy to accept” [23].

All participants were requested to report Adverse events (AEs) in the study during the treatment and follow-up periods. The severity (divided into mild, moderate, and severe) of AEs was evaluated by the investigator.

### Calculation of sample size and statistical analyses

The sample size was calculated based on the mean SIT value. According to our pilot trial [16], the increase in the mean SIT value in the acupuncture group at week 8 was 3.41 ± 3.04. A previous report indicated a mean increase in the SIT value in an artificial tear group of 1.8 ± 1.6 [24]. Based on these values, a sample size of 120 was needed to detect a difference of 1.61 between the two groups, which provided 90% power with a two-sided 5% level of significance, considering a 20% dropout rate.

SPSS Version 26.0 software was used for statistical analysis. All statistical analyses used bilateral tests. P values less than 0.05 were considered to be statistically significant. The analysis of efficacy was based on the principle of intentionality analysis. All participants who had at least one efficacy assessment were all included in the efficacy analysis. A sensitivity analysis was performed considering the loss during the study. The missing data was replaced by using the last observation carried forward method [25].

For data with a natural or adjusted normal distribution, we used mean and 95% confidence interval (CIs). For data with a non-normal distribution, we used the median and 95% CI. Group *t*-tests or non-parametric rank and sum tests were used for between-group comparisons. For within-group comparisons to baseline values, *t*-tests or non-parametric rank and sum tests were used. Independent sample *t*-tests were used to compare different time-points. Categorical variables were described using composition ratios and/or 95% confidence intervals. Chi-square test or Fisher’s exact test was used for comparisons between groups.

## Results

### Enrollment

**Fig. 2:**
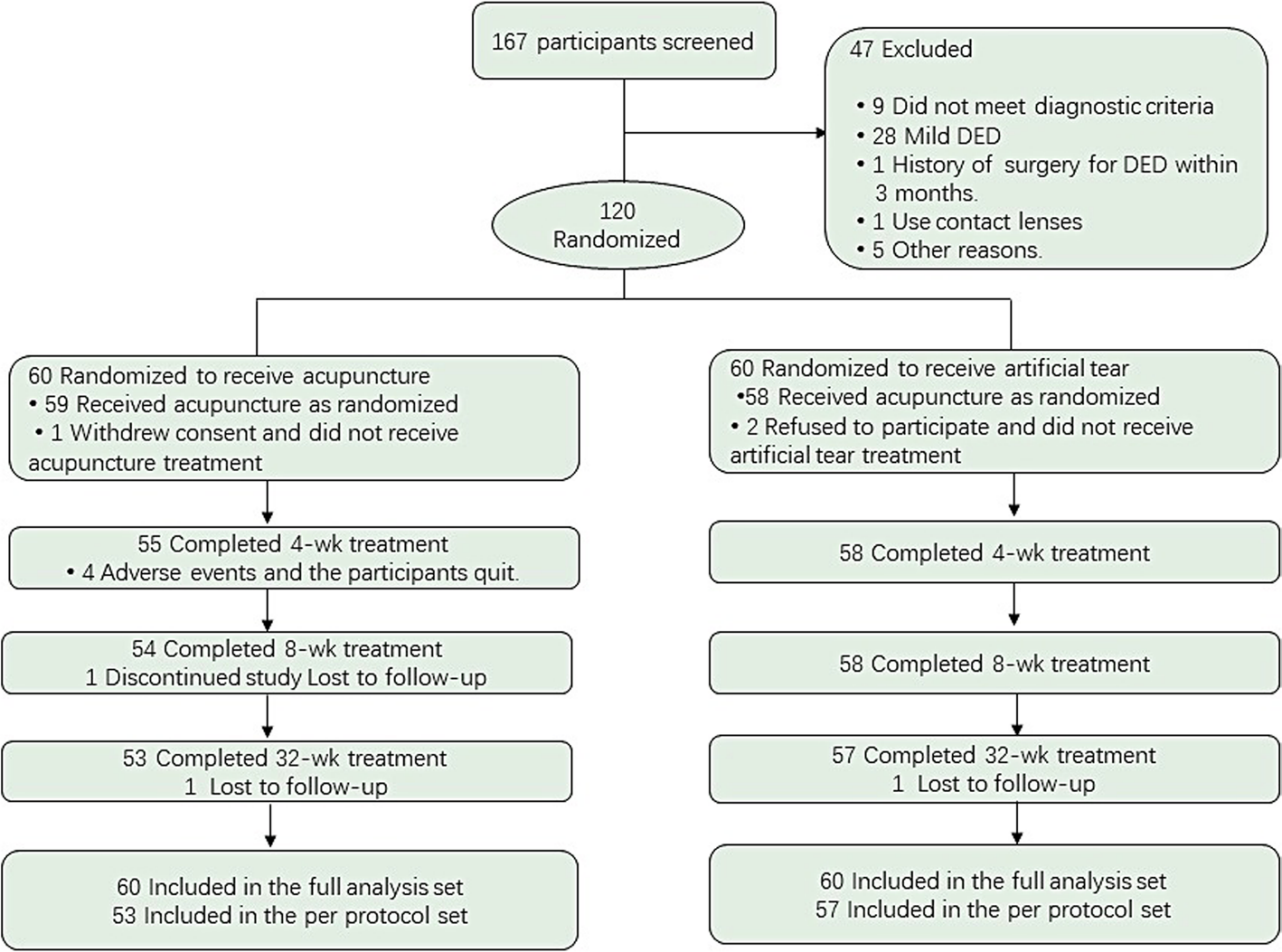
Flow of participants

We screened 167 participants with DED for eligibility, and excluded 47; thus, 120 participants were assigned to either the acupuncture group (*n* = 60) or the AT group (*n* = 60), at a ratio of 1:1. Among them, 113 (94.2%) participants completed at least 4-week treatments and 110 (91.7%) participants completed all the trials (Fig. 2). Baseline characteristics are shown in Table 1; these were similar between the groups.

**Table 1:** Participant baseline characteristics^a^

Characteristic	Acupuncture group (*n*=60)	Artificial tear group (*n*=60)	*P* value
Age (years) (mean, 95%CI)	44.16 (35.54 to 49.43)	41.28 (31.78 to 45.78)	0.224
Sex, M/F (No.)	22/38	29/31	0.437
Educational level (No. [%])			
Primary education or less	16 (26.7)	11 (18.3)	0.246
Secondary education	38 (63.3)	42 (70.0)	0.413
Tertiary education	6 (10.0)	7 (11.7)	0.421
DED duration, median (IQR), months	26 (6–60)	22 (5–60)	0.249
Profession (No. [%])			
Office worker	30 (50.0)	34 (56.7)	0.341
Physical work	12 (20.0)	11 (18.5)	0.512
Retired or unemployed	18 (30.0)	15 (25.0)	0.218
Comorbidities (No. [%])^**b**^			
Cardio vascular and cerebro vascular diseases	6 (10.0)	4 (6.7)	0.362
Metabolic disorders	5 (8.3)	6 (10.0)	0.438
Eye conditions	9 (15.0)	7 (11.7)	0.327
Other	2 (3.3)	3 (5.0)	0.357
Sleep disorders (No. [%])	3 (5.0)	3 (5.0)	0.553
SIT	4.37 (3.37 to 5.25)	3.82 (2.84 to 4.86)	0.255
Average symptom NRS (mean, 95%CI)	7.14 (4.29 to 8.85)	6.83 (4.27 to 8.63)	0.316
OSDI (mean, 95%CI)	34.50 (29.15 to 41.23)	32.20 (27.80 to 40.12)	0.426
TBUT (mean, 95%CI)	4.31 (3.38 to 5.25)	4.40 (3.56 to 5.32)	0.418
CFS (mean, 95%CI)	3.25 (2.93 to 3.62)	2.80 (2.56 to 3.08)	0.227

*SD* standard deviation, *DED* dry eye disease, *SIT* Schirmer-I test, *NRS* numerical rating scale, *OSDI* ocular surface disease index, *TBUT* tear break-up time, *CFS* corneal fluorescence staining, *IQR* inter-quartile range

^a^There were no significant between-group differences at baseline

^b^Two participants in the acupuncture group and one participant in the artificial tear group had no the records of complications and concomitant medication

### Missing data and dropouts

Adherence to acupuncture treatments (3 times per week for 8 weeks) or AT treatments (4 times per day for 8 weeks) was similar in both groups, 90.0% and 96.7%, respectively. Of the 120 patients, 113 patients completed 4-week treatments and 110 patients completed all treatments and follow-ups. The data of the 120 participants was analyzed in the full analysis set based on the intentionality analysis principle. A sensitivity analysis was performed in the per-protocol set, which included the participants that completed all the treatments and follow-ups. The original results in this study were stable and reliable based on the sensitivity analysis.

### Outcomes

#### Primary outcome measures

For the primary outcome (Table 2), the mean SIT value was 4.37 (95%CI, 3.37–5.25) at baseline and 9.25 (95%CI, 6.00–12.75) at week 8 in the acupuncture group, and 3.82 (95%CI, 2.84–4.86) at baseline and 4.34 (95%CI, 2.94–5.98) at week 8 in the AT group. The increase in the SIT value at week 8 was greater in the acupuncture group (mean, 5.75) than in the AT group (mean, 0.52), with a mean difference of 5.23 (95%CI, 1.34–9.12; *P* = 0.01).

**Table 2:** Primary and secondary outcomes^a^

Variable	Acupuncture group (*n*=60)	Artificial tear group (*n*=60)	Difference (95% CI)	*P* value
**Primary outcome**				
*SIT at weeks 8, mean (95% CI)*	9.25 (6.00 to 12.75)	4.34 (2.94 to 5.98)		
*SIT Change, at weeks 8, mean (95%CI)*	5.75 (2.53 to 9.75)	0.52 (− 1.18 to 2.46)	5.23 (1.34 to 9.12)	0.01
**Secondary outcomes**				
*Change, SIT, mean (95% CI)*				
At week 4	5.16 (2.94 to 7.87)	− 0.30 (− 1.62 to 1.28)	5.46 (2.73 to 8.18)	<0.001
At week 32	2.05 (0.94 to 4.47)	0.16 (− 1.94 to 2.83)	1.89 (0.54 to 4.07)	0.066
*Change, Average symptom NRS*^a^*, mean (95% CI)*				
At week 4	− 2.26 (− 5.07 to1.63)	− 1.69 (− 5.05 to 1.89)	0.57 (− 2.15 to 2.46)	0.162
At week 8	− 2.96 (− 4.94 to 1.87)	− 2.55 (− 4.25 to 1.95)	0.38 (− 2.46 to 3.08)	0.084
At week 32	− 3.10 (− 2.46 to1.54)	− 1.22 (− 0.89 to 0.09)	1.88 (− 0.46 to 3.57)	0.036
*Change, OSDI, mean (95% CI)*^b^				
At week 4	− 9.46 (− 32.66 to 13.59)	− 5.26 (− 20.05 to 21.89)	4.20 (− 14.35 to 18.56)	0.140
At week 8	− 16.14 (− 35.40 to 16.30)	− 7.65 (− 19.25 to 15.05)	8.49 (− 5.26 to 22.14)	0.036
At week 32^a^	− 18.18 (− 26.46 to 16.54)	− 6.03 (− 21.89 to 19.32)	12.15 (− 4.68 to 15.46)	0.017
*Change, TBUT, mean (95% CI)*				
At week 4	1.56 (− 0.13 to 3.06)	0.16 (− 0.80 to 1.16)	1.40 (− − 0.46 to 3.27)	0.136
At week 8	1.72 (−.12 to 3.25)	0.44 (− 0.76 to 1.68)	1.28 (− 1.01 to 3.26)	0.294
At week 32	0.46 (− 0.14 to 2.46)	0.12 (−0.43 to 1.89)	0.34 (− 1.01 to 2.26)	0.569
*Change, CFS, mean (95% CI)*				
At week 4	− 2.99 (1.00 to 4.25)	− 2.38 (0.40 to 3.96)	0.61 (− 0.18 to 2.54)	0.136
At week 8	− 3.25 (0.98 to 4.58)	− 3.06 (0.78 to 4.14)	0.29 (− 0.85 to 2.12)	0.214
At week 32	− 2.15 (1.54 to 2.46)	− 2.57 (− 0.09 to 0.89)	0.14 (− 1.07 to 1.92)	0.325
*Treatment acceptability assessment, number(% )*^c^	Little difficult: 6 (10%) Moderate: 16 (26.7%) Easy: 32 (53.3%) Very easy: 6 (10.0%)	Little difficult: 5 (8.3%) Moderate: 10 (16.7%) Easy: 35 (58.3%) Very easy: 10 (16.7%)		0.130

*SIT* Schirmer-I test, *NRS* numerical rating scale, *OSDI* ocular surface disease index, *TBUT* tear break-up time, *CFS* corneal fluorescein staining, *CI* confidence intervals

^a^Scoring is the sum of the totals divided by 8 (8 symptoms in total)

^b^Scoring was additive (0 [better outcomes] to 44 [worse outcomes]). Minimal clinically important difference: 13.9

^c^Treatment acceptability was assessed by the chi-square tests. All participants (120) were included in the acceptability assessment and the participants who did not complete the treatments were all evaluated as a little difficulty in acceptability

Similar results were observed at week 4 (Table 2). The increase in the SIT value at week 4 was greater in the acupuncture group (mean, 5.16) than in the AT group (mean, − 0.3) with a mean difference of 5.46 (95%CI, 2.73–8.18; *P* < 0.001). However, the SIT values were not significantly different between both groups at week 32.

#### Secondary outcome measures

No significant differences in the change in mean DED symptom NRS scores were found between the groups (all *P* > 0.05). Symptoms varied among participants, and not all participants experience every listed symptom. The acupuncture group had significantly decreased NRS scores for eye dryness, eye pain, and blurred vision compared to the AT group at multiple time-points. There was no significant difference between both groups for other symptoms.

The acupuncture group showed a more significant decrease than the AT group in the OSDI score at weeks 8 (8.49 [95%CI, − 5.26–22.14]) and 32 (12.15 [95%CI, − 4.68–15.46]) (all *P* < 0.05), but not at week 4 (*P* > 0.05).

There were no significant differences in the change in TBUT between both groups. A minor difference of 1.4 (95%CI, − 0.46–3.27) was detected at week 4, 1.28 (95%CI, − 1.01–3.26) at week 8, and 0.34 (95%CI, − 1.01–2.26) at week 32. The differences between both groups were not significant (all *P* > 0.05).

There were no significant differences in the change in CFS from baseline to all time-points between both groups. The reduction in CFS was similar between both groups.

Acupuncture and AT acceptability were assessed after 8 weeks of intervention. 63.3% of participants in the acupuncture group and 75.0% in the AT group is easily acceptable or very easily acceptable. No significant differences were observed between the groups (*P* = 0.13). The detailed data are shown in Table 2.

### Safety outcomes

Treatment-related AEs occurred in 8.3% of participants in the acupuncture group; AEs included mild bleeding, which was absorbed quickly, or moderate bleeding, which was resolved by cold compress repeatedly for 24 h, and there were no other AEs. Common termination criteria adverse events (CTCAE) were used to evaluate the level of use in the study. AEs were divided into five grades according to CTCAE, in which 3 participants were grade one and 2 participants were grade two. To avoid the occurrence of AEs related to acupuncture, acupuncturists were advised to slow down the frequency of acupuncture manipulation and routinely use cold compress for a while after acupuncture, which may reduce the occurrence of bleeding related to AEs. Incidence of AEs in the AT group was 3.4%, which included nausea (1 participant) and dizziness (1 participant). However, it was unclear whether these AEs were related to the treatment. No other has occurred (Table 3).

**Table 3:** Adverse events

Adverse event	Participants (No. [%])	
	Acupuncture group (*n*=60)	Artificial tear group (*n*=60)
Overall	5 (8.3)	2 (3.4)
Bleeding (mild)	3 (5)	0
Bleeding (moderate)	1 (1.7)	0
Sharp pain	1 (1.7)	0
Feel nauseated	0	1 (1.7)
Dizziness	0	1 (1.7)

Adverse events were analyzed in all participants who received treatment. Adverse events were counted by type other than the frequency in the same participant. Adverse events with different types occurring in a single participant were defined as independent adverse events. An adverse event with multiple occurrences in a single participant was defined as 1 adverse event

## Discussion

In this RCT, we compared acupuncture and ATs for DED. After 4 and 8 weeks of treatment, acupuncture resulted in greater improvement in the SIT value than AT. OSDI score change in the acupuncture group was also significantly better than AT group. However, between-group differences of the changes in the average symptom NRS score, TBUT, and CFS did not differ significantly at week 8. The overall incidence of AEs was low.

The SIT value provides direct objective evidence for the therapeutic value of acupuncture. In this study, the SIT value in the acupuncture group increased by 5.16 and 5.75 after 4 and 8 weeks, respectively. Previous studies showed an increase of 0.41 [12] and 0.96 [13], which was significantly lower than that in our study. The acupuncture method used in this study may have been more conducive to promoting tear secretion than those used in previous studies, due to the acupoint used, the stronger and longer duration of needling manipulation, and the tear-flow method after needling manipulation.

The OSDI is also an important indicator of DED symptoms. In this trial, the change in the OSDI score in the acupuncture group (Table 2) was consistent with previous findings. In our study, the OSDI score decreased by 16.14 after 8 weeks in the acupuncture group, similar to the decrease of 16.11 found previously [12]. A median reduction of 13.9 in the OSDI score represents the minimal clinically important difference [26].

Although previous studies have shown that acupuncture can improve DED symptoms, there were some differences in findings. In our study, acupuncture significantly improved the symptoms of dry eye, itching, eye pain, and blurred vision; however, it was less effective in improving other DED symptoms (photophobia, blurred vision, visual fatigue, and foreign body sensation) than AT, possibly due to the acupuncture point used, needle manipulation, and inclusion of moderate to severe DED participants. In this study, acupuncture stimulated lacrimal secretion and improved lacrimal gland function, rapidly relieving many DED symptoms in a sustained manner. A previous study showed that acupuncture could release opioid peptides, which may explain the relief of eye pain by acupuncture [27]. Acupuncture also altered the tear composition and regulated the immune system, thereby reducing inflammation or an allergic reaction at the eye surface [28].

In this study, objective indicators other than the SIT value did not improve significantly. A previous study on DED showed that the TBUT increased significantly, by 0.95, after acupuncture, but not after sham acupuncture [29]. Another study showed that TBUT increased by 1.93 after acupuncture treatment [13].

Participants in both groups generally found their treatment acceptable. The use of a single acupoint, rather than multiple acupoints as in previous studies, reduced the pain of acupuncture, possibly contributing to its acceptability.

Most previous studies showed that acupuncture treatment only improved subjective DED assessments, but did not investigate objective assessments or did not find significant improvement in the objective indicators (SIT, TBUT, CFS) [30, 31]. In this study, acupuncture treatment at BL1 with needle manipulation resulted in approximately 10 times more tear secretion than in the AT group, suggesting the mechanism underlying the efficacy of acupuncture. The BL1 point is located on the lacrimal caruncle of the inner canthus, and the tissues in the lacrimal caruncle and Krause glands may come in contact during acupuncture, which could regulate basic tear secretion and help maintain ocular surface homeostasis. When acupuncture was performed in the nasal direction, the sensory neurons in the lacrimal reflex arc may have been stimulated, thus triggering reflexes through the trigeminal nerve, which were transmitted to the lacrimal gland to promote tear production and regulate tear secretion [32].

The anatomical position of BL1 may be related to the better curative effect observed in many previous acupuncture trials for ophthalmic diseases [33]. Modern neuroanatomy indicates that the BL1 point belongs to the trigeminal nerve distribution. When the BL1 point is stimulated, the trigeminal nerve could produce excitatory signals in the brain, which could improve visual function.

This study had some limitations. First, we did not limit the subtype of DED when including study participants. Our findings may be more relevant to the lacrimal deficiency type. Second, because the AT group was the control group not performed blinding, which may have affected the study results. The environment of the treatment room and the repeated health education of acupuncturists may give participants some positive psychological hints, which may contribute to improving the effect. Thirdly, the effect of acupuncture may be related to the psychological expectation of the subjects before treatment. This should be investigated in the future.

Based on these limitations, stratification of participants can be added to trials in the future. Acupuncture may have a better effect on DED with water deficiency than other types of DED, as it can promote tear secretion. Once there are many significant differences between groups on the types of DED in the included participants, the results will be biased. The placebo control group may be set up in the trials in the future, and an appropriate placebo group which can not be recognized by participants will be needed. Finally, expectations of the participants should be investigated before the trial in the future.

## Conclusions

Acupuncture at the BL1 point alone can significantly improve lacrimal secretion and symptoms of moderate and severe DED, with a better curative effect than that of AT. Acupuncture treatment was acceptable to patients and there were no serious AEs. Thus, this simple acupuncture treatment could be considered safe, effective, and acceptable for moderate to severe DED.

## Supplementary Information

**Additional file 1: Supplement 1.** Trial Protocol and Statistical Analysis Plan.

## Data Availability

The full data set will be made available. Requests for the data to be released should be sent to ZWZ (principal investigator).

## Abbreviations

DED: Dry eye disease
AT: Artificial tear
SIT: Schirmer-I test
NRS: Numerical rating scale
OSDI: Ocular surface disease index
TBUT: Tear break-up time
CFS: Corneal fluorescein staining
RCT: Randomized controlled trial
AEs: Adverse events
CI: Confidence interval
DEWS: Dry Eye Work Shop
ITT: Intention-to-treat
SD: Standard deviation
SPIRIT: Standard Protocol Items: Recommendations for Interventional Trials
STRICTA: Standards for Reporting Interventions in Clinical Trials of Acupuncture
CTCAE: Common termination criteria adverse events
