# Supplementary material for: Effectiveness of acupuncture at acupoint BL1 (Jingming) in comparison with artificial tears for moderate to severe dry eye disease: a randomized controlled trial
Source: Trials. 2022 Jul 27;23:605. doi: 10.1186/s13063-022-06486-4 (PMC9327344; doi:10.1186/s13063-022-06486-4)
Supplement: Supplementary file 1 — Additional file 1: Supplement 1. Trial Protocol and Statistical Analysis Plan. [file 13063_2022_6486_MOESM1_ESM.pdf]

Trial Protocol and Statistical Analysis Plan

1. Study design

This is a two-center, prospective, controlled, randomized trial conducted in patients with moderate to severe DED. The flow chart is shown in Figure 1. We have developed the design and protocol based on the guidance for the Standard Protocol Items: Recommendations for Interventional Trials (SPIRIT) and the Standards for Reporting Interventions in Clinical Trials of Acupuncture (STRICTA).[1, 2]

2. Study setting and recruitment

The study will commence on September 1, 2018, and will be completed by December 31, 2020, at the Guang'anmen Hospital, China Academy of Chinese Medical Sciences and the South Area of Guang'anmen Hospital, China Academy of Chinese Medical Sciences. DED is diagnosed according to the criteria of International DEWS by an experienced ophthalmologist. [3] The participants identified as eligible will be randomized into two groups—acupuncture and medicine—at a ratio of 1:1. The flowchart of the trial is shown in Figure 1. The total study period for this trial is 32 weeks (Figure 2), including 8-week treatment period and 24-week follow-up period.

3. Inclusion criteria

The inclusion criteria will be as follows.

(1) Individuals will be more than 18 years of age and less than 60 years of age.

(2) Participants will meet the diagnostic criteria of DED.[3] They were required to have one or more symptoms of DED (e.g. eye dryness, foreign body sensation, itching, burning, stinging, visual fatigue, photophobia or blurred vision) with severity of ≥4 (range: 0–10) measured by numerical rating scale (NRS). a Schirmer-I test (SIT) (with the application of cocaine, a local anesthetic) value < 10mm/5minutes, a tear film break-up time (TBUT) < 10 seconds, and corneal fluorescein staining (CFS) scale ≥ 1. [4]

(3) Only the single eye with more severe scores or symptoms will be selected to observe the outcome.

(4) Participants who can complete the study, treatment, and assessments will be selected.

4. Exclusion criteria

The exclusion criteria will be as follows:

(1) participants with other ocular diseases (e.g., lacrimal duct obstruction, other conjunctiva and keratopathy) or immune system diseases (e.g., Sjogren syndrome and rheumatoid arthritis);

(2) participants with a history of surgery for DED within 3 months;

(3) participants with cardiovascular, kidney, liver, or hematopoietic system diseases, mental health disorders, cancer or other severe diseases for whom acupuncture may be inappropriate or unsafe;

(4) participants who received acupuncture within the past week;

(5) women who are pregnant or lactating;

(6) individuals who are participating in other clinical trials; and

(7) participants with a pacemaker, metal allergy, or severe fear of needles.

5. Randomization and blinding

The randomization will be performed by the Drug Clinical Trial Office affiliated with Guang'anmen Hospital using a centralized computer system. Patients were randomly assigned to the acupuncture or AT group using a block size of 4 stratified by centers, the final group assignments were placed in 120 opaque sealed envelopes, which were serially numbered and kept by a researcher not involved in treatment procedures or data analysis. After informed consent was obtained, the researcher opened the envelope according to the order in which the participant entered the trial and was provided for the prescribed treatment. All participants received either acupuncture or ATs 3 times per week over the 8-week period, and both groups were followed up for another 24 weeks.

6. Data collection

The data collection will be from the case report forms recorded by the investigator in the trial. The demographic, clinical, and examination results of DED from the

participants will be recorded at baseline and during the treatment and follow-up periods. Moderate to severe DED will be diagnosed by an experienced ophthalmologist after an ophthalmology examination. The investigator will enter all of the collected data into the case report forms. During the treatment period (8th week) and the follow-up period (32nd week), the forms concerning the questionnaire survey will be completed by the participants under the guidance of a full-time staff member; other forms concerning the ophthalmology examination will be filled in by the investigator according to objective examination results.

7. Quality control

The acupuncture practitioners responsible for the acupuncture treatments must have a minimum of 5 years of clinical experience. All of the investigators will take a one-day training course for this trial. This training course will cover the study protocol, acupuncture operation, precautions for drug use, the recording method for CRF, disposal of bleeding, a researcher duties and research monitoring. Full-time researchers who are not aware of the random allocation of groups will be responsible for the assessments of all scales. During the trial, supervisors will check on case reports and acupuncture treatments once a month. Drop-outs, withdrawals (with the reasons), and the compliance of all participants will be recorded in detail throughout the treatment and follow-up period. Two postgraduates (not involved in the trial) will input the data independently and verify the database together.

8. Ethics and dissemination

The study protocol was in accordance with the principles of the Declaration of Helsinki, [5] was approved by the Ethics Committee of Guang'anmen Hospital of China Academy of Chinese Medical Sciences (approval number 2018-012-KY-01) on April 19, 2018 and was registered on April 23, 2018, at http://www.chictr.org.cn/ (ref. ChiCTR1800015831). All important protocol modifications will be submitted to the Ethics Committee of Guang'anmen Hospital. The researchers will inform all participants of the potential risks and benefits of the study. Informed consent will be

obtained for all participants from either the participant or the participant's legal representative. All participants will be required to be able to understand written instructions, complete the examination and assessment forms. The personal information of all participants will be hidden from other users and stored in a specific cabinet with a lock. Statistics and monitoring managers will have the right to obtain the final study data set. To prevent the participants from suffering harm from trial participation, we will provide corresponding treatment and medical consultations with the ophthalmologist for free. We will disseminate the results of this study in meetings or publications when the trial is completed.

9. Statistical analysis

SPSS Version 26.0 (International Business Machines Corporation, China) software will be used to perform the statistical analysis. Two-sided tests will be used for all statistical analyses. The level of significance is established at 0.05. Efficiency and safety analyses will be based on all participants at randomization according to the intention-to-treat (ITT) principle. All participants who have accepted randomization will be included in the statistical analysis of data. All original data collected from participants in the trial will be included in the full statistical analysis set. The data cannot be changed or eliminated at random. The missing data in the case report forms will be replaced by using the last observation carried forward method. [6]We will do sensitivity analyses in the per protocol set, which included all randomised patients who had no major protocol deviation.

We will use the mean and standard deviation (SD) to describe continuous data if the data have a normal distribution and the mean and 95% confidence interval (CI) to describe continuous data if the data are skewed. We will use the percentage or 95% CI to describe categorical data. For comparisons with baseline data, we will use a paired *t*-test for continuous data and a nonparametric test for categorical data. For comparisons of the differences between the two groups, T-tests or Mann–Whitney U tests will be used for comparisons between continuous variables, and chi-square tests or Fisher's exact tests will be used for comparisons between categorical variables, as appropriate.

For assessing safety in the trial, the investigator will present the AEs and adverse reactions with tables, which will comprise the categories of AEs, the severity of AEs, the incidence of AEs, and correlation with the treatment. Every participant with serious AEs will stop receiving the intervention. All details will be reported in a timely manner.

References

1. Chan AW, Tetzlaff JM, Gotzsche PC, et al. SPIRIT 2013 explanation and elaboration: guidance for protocols of clinical trials. *BMJ (Clinical research ed)* 2013, 346:e7586.
2. MacPherson H, Altman DG, Hammerschlag R, et al. Revised STandards for Reporting Interventions in Clinical Trials of Acupuncture (STRICTA): Extending the CONSORT statement. *Journal of evidence-based medicine* 2010, 3(3):140-155.
3. Thulasi P, Djalilian AR. Update in Current Diagnostics and Therapeutics of Dry Eye Disease. *Ophthalmology* 2017, 124(11s):S27-s33.
4. Robert PY, Cochener B, Amrane M, et al. Efficacy and safety of a cationic emulsion in the treatment of moderate to severe dry eye disease: a randomized controlled study. *Eur*opean journal of ophthalmology 2016; 26(6):546-555.
5. World Medical Association Declaration of Helsinki: ethical principles for medical research involving human subjects. *The Journal of the American College of Dentists* 2014, 81(3):14-18.
6. Zhao L, Chen J, Li Y, etal . The long-term effect of acupuncture for migraine prophylaxis: a randomized clinical trial. *JAMA Intern Med 2017*;177:508-15. 10.1001/jamainternmed.2016.9378 28241154

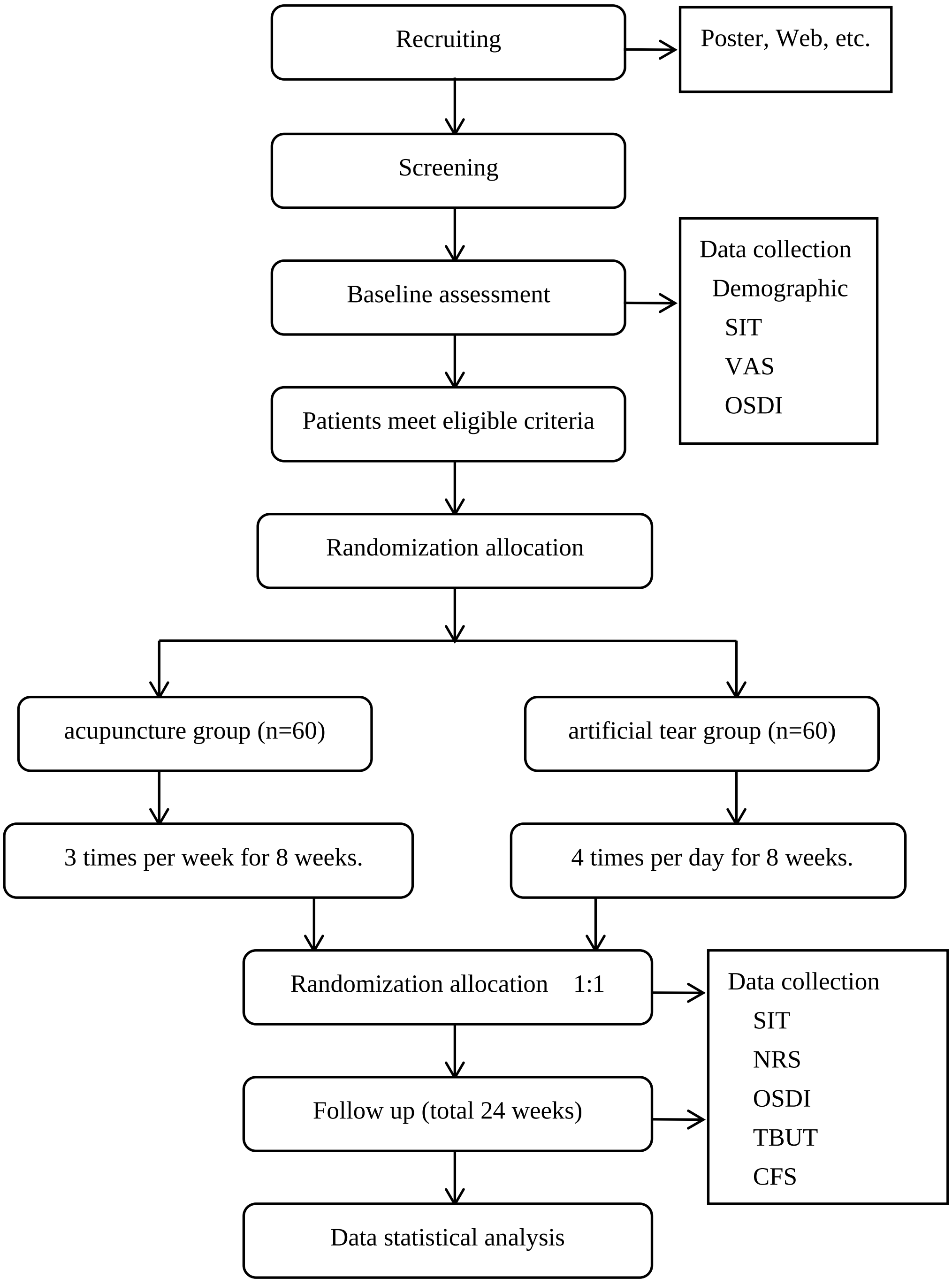

Figure 1 Diagram of trial design. SIT, Schirmer-I test; TBUT, tear film breakup time; CFS, corneal fluorescein staining; OSDI, ocular surface disease index ; VAS, visual analogue score.

	STUDY PERIOD				
	Enrolment	Allocation	Post-allocation		
TIME POINT (week)	-1	0	4	8	32
ENROLMENT					
Eligibility screen	×				
Informed consent	×				
Demographics	×				
Allocation		×			
INTERVENTIONS:					
Acupuncture			——	——	
Artificial tear			——	——	
ASSESSMENTS:					
SIT		×	×	×	×
NRS		×	×	×	×
OSDI		×	×	×	×
TBUT		×	×	×	×
CFS		×	×	×	×
Acceptance				×	

Figure. 2 Standard protocol items: recommendation for interventional trials (SPIRIT) schedule of enrolment, interventions, and assessments. SIT, Schirmer-I test; VAS, visual analogue score; OSDI, ocular surface disease index ; TBUT, tear film breakup time; CFS, corneal fluorescein staining.
